# Senataxin: A New Guardian of the Female Germline Important for Delaying Ovarian Aging

**DOI:** 10.3389/fgene.2021.647996

**Published:** 2021-04-29

**Authors:** Hayden A. Homer

**Affiliations:** The Christopher Chen Oocyte Biology Research Laboratory, UQ Centre for Clinical Research, The University of Queensland, Herston, QLD, Australia

**Keywords:** premature ovarian aging, premature ovarian insufficiency, ovarian aging, ovarian reserve, oocyte, DNA damage, oocyte quality, oxidative stress

## Abstract

Early decline in ovarian function known as premature ovarian aging (POA) occurs in around 10% of women and is characterized by a markedly reduced ovarian reserve. Premature ovarian insufficiency (POI) affects ~1% of women and refers to the severe end of the POA spectrum in which, accelerated ovarian aging leads to menopause before 40 years of age. Ovarian reserve refers to the total number of follicle-enclosed oocytes within both ovaries. Oocyte DNA integrity is a critical determinant of ovarian reserve since damage to DNA of oocytes within primordial-stage follicles triggers follicular apoptosis leading to accelerated follicle depletion. Despite the high prevalence of POA, very little is known regarding its genetic causation. Another little-investigated aspect of oocyte DNA damage involves low-grade damage that escapes apoptosis at the primordial follicle stage and persists throughout oocyte growth and later follicle development. Senataxin (SETX) is an RNA/DNA helicase involved in repair of oxidative stress-induced DNA damage and is well-known for its roles in preventing neurodegenerative disease. Recent findings uncover an important role for SETX in protecting oocyte DNA integrity against aging-induced increases in oxidative stress. Significantly, this newly identified SETX-mediated regulation of oocyte DNA integrity is critical for preventing POA and early-onset female infertility by preventing premature depletion of the ovarian follicular pool and reducing the burden of low-grade DNA damage both in primordial and fully-grown oocytes.

## Introduction

Senataxin (Setx) is an RNA/DNA helicase required for multiple DNA processes including transcriptional regulation, the resolution of RNA:DNA hybrids (or R-loops) arising at transcription pause sites and DNA repair (Lavin et al., 2013; Groh et al., 2017). Regarding DNA repair, Setx is especially critical for promoting the repair of DNA damage induced by increased oxidative stress (Suraweera et al., 2007).

In humans, *SETX* mutations cause the autosomal recessive neurodegenerative disorder, ataxia with oculomotor apraxia type-2 (AOA2). AOA2 belongs to a group of rare autosomal recessive cerebellar ataxias (ARCAs), which also include Friedreich ataxia and ataxia-telangiectasia (A-T; Palau and Espinós, 2006). AOA2 is characterized primarily by cerebellar atrophy with prominent gait ataxia, a peripheral sensorimotor neuropathy, areflexia, and elevated levels of α-fetoprotein. Onset of symptoms is usually early, occurring between 10 and 20 years of age (Lavin et al., 2013). Interestingly, *SETX* mutations may also cause up to three other neurological conditions including the less common autosomal dominant neurodegenerative disorder, juvenile amyotrophic lateral sclerosis (ALS4), a form of juvenile ALS characterized by limb weakness, severe muscle wasting, normal sensation, and pyramidal tract signs (Lavin et al., 2013). Intriguingly, therefore, different mutations in the same *SETX* gene can give rise to several distinct disorders depending on their nature and localization.

Separate from its role in neurological disorders, more recent research has begun to shed light on a lesser-known role of Setx in reproduction (Becherel et al., 2013, 2019; Subramanian et al., 2020, 2021). Setx has been shown to be critical for male fertility through maintaining genomic integrity during spermatogenesis (Becherel et al., 2013). This arises because DNA double-strand breaks (DSB) induced during the process of reciprocal recombination remains unrepaired in the absence of Setx thereby activating a pachytene checkpoint that eliminates spermatocytes (Becherel et al., 2013). This function is conserved in humans as spermatogenesis in men with AOA2 is severely compromised and associated with persistent DNA breaks in spermatocytes (Becherel et al., 2019). Notably, there have been case reports of compromised reproductive function in females with AOA2 (Lynch et al., 2007; Gazulla et al., 2009) suggesting that *SETX* may also be important for ovarian function. Here, I review very recent data from the mouse model revealing that Setx is important for protecting DNA integrity in oocytes but unlike males, is dispensable for oogenesis and exhibits a unique role in slowing ovarian aging.

## Vulnerability of Oocytes to Oxidative Stress and DNA Damage During Aging

**Figure 1:**
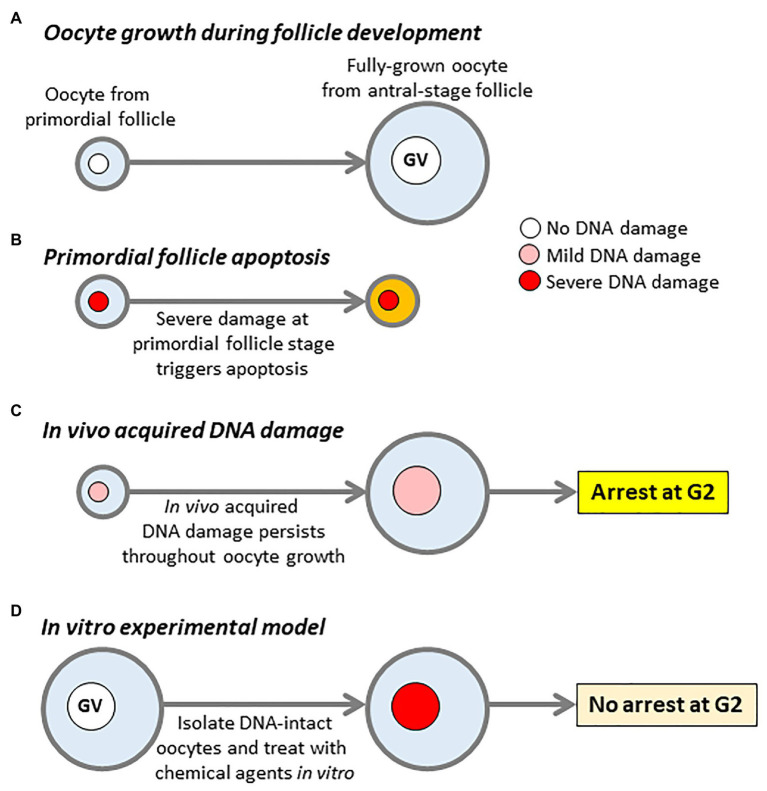
DNA damage models and impact of DNA damage on oocytes. **(A)** Oocyte growth during follicle development. Oocytes undergo an extensive period of growth, while follicles develop from primordial to antral stages. Large antral-stage follicles contain fully-grown oocytes arrested at first meiotic prophase, identifiable by the presence of an intact germinal vesicle (GV). **(B)** Oocytes within primordial follicles, which sustain severe DNA damage trigger a TAp63-dependent pathway resulting in follicular apoptosis. **(C)** DNA damage sustained *in vivo* which does not trigger primordial follicle apoptosis persists throughout oocyte growth resulting in damaged fully-grown oocytes within antral-stage follicles. **(D)** The canonical model for studying the effects of DNA damage in fully-grown oocytes. Fully-grown oocytes with intact DNA are isolated from young female mice and subject to acute treatment with agents such as chemotherapy drugs to induce severe levels of damage. Note that the key differences between *in vivo* damage **(C)** and the *in vitro* experimental model **(D)** are, firstly, *in vivo* damage is much milder than *in vitro*-induced damage and, secondly, *in vivo* damage persists for weeks to months during oocyte growth vs. hours in the case of the *in vitro* model.

Females are born with a finite complement of germ cells, which diminish throughout postnatal life becoming almost completely exhausted by the time of the menopause (Oktem and Urman, 2010; Wallace and Kelsey, 2010). During fetal life, germ cells become surrounded by flattened somatic cells forming primordial follicles, which constitute the most abundant follicle-type in the ovary (Oktem and Urman, 2010). The ovarian reserve of oocytes is therefore defined by the primordial follicle reservoir. Cohorts of primordial follicles undergo spontaneous activation and thereafter progress over a period of 2–3 months in humans (2–3 weeks in mice) through primary, secondary, and antral stages of development concomitantly with growth of their contained oocytes (Figure 1A; Oktem and Urman, 2010; Greaney et al., 2018). Within follicles, oocytes are arrested at the dictyate stage of prophase of the first meiotic division (Oktem and Urman, 2010; Greaney et al., 2018). Since primordial follicles are laid down before birth, some oocytes remain arrested for up to 4–5 decades in humans before resuming development.

Cellular oxidative stress is harmful to key cellular components and levels increase with aging (Lim and Luderer, 2011). In keeping with this, oocytes from older females exhibit increased levels of reactive oxygen species (ROS; Subramanian et al., 2020). Increased oxidative stress is therefore considered a leading cause of the age-related decline in oocyte quality (Tarin, 1996; Kasapoğlu and Seli, 2020; Homer, 2021). Since mitochondrial oxidative phosphorylation is the major source of cellular ROS, mitochondrial dysfunction is a focal point of oocyte aging (May-Panloup et al., 2016; Homer, 2020, 2021; Kasapoğlu and Seli, 2020).

Recent *in vivo* findings reinforce the critical importance of oxidative stress in female reproductive aging. Levels of the essential cellular co-factor, nicotinamide adenine dinucleotide (NAD^+^), were shown to decline with aging in oocytes and, importantly, restoring oocyte NAD^+^ reserves by feeding of an NAD^+^ precursor could rejuvenate oocytes and restore fertility in older females (Bertoldo et al., 2020). NAD^+^ in turn regulates oxidative stress in oocytes at least in part *via* a family of NAD^+^-dependent deacetylases and deacylases known as sirtuins (SIRT1-7); oocytes from mice over-expressing SIRT2 exhibit delayed aging associated with reduced ROS (Bertoldo et al., 2020), while deletion from oocytes of another sirtuin, SIRT1, results in increased oxidative stress that compromises embryonic development leading to early-onset female infertility (Iljas et al., 2020). Interestingly, the importance of NAD^+^ for oocyte quality extends beyond sirtuin-support roles as it is also critical for enabling oocytes to retain maximum cytoplasmic reserves during meiotic division (Wei et al., 2020). It has also been found that expression of enzymes responsible for producing coenzyme Q10 (CoQ10), a major antioxidant and component of the mitochondrial electron transport chain, is reduced in oocytes from aged mouse and human oocytes (Ben-Meir et al., 2015). Moreover, supplementation of aging female mice with CoQ10 markedly improved oocyte developmental competence thereby boosting fertility (Ben-Meir et al., 2015).

Since oxidative stress is known to induce DNA damage (Suraweera et al., 2007; Subramanian et al., 2020), progressive mitochondrial deterioration and increasing ROS leaves oocytes uniquely vulnerable to DNA damage during their protracted dormancy in ovaries. In line with this, oocytes from older female mice and women exhibit increased levels of DNA DSBs (Titus et al., 2013).

## Two Fates for Oocytes Following *in Vivo* Acquired DNA Damage

Extensive levels of DSBs in primordial-stage oocytes secondary to severe chemotherapy/irradiation-induced injury in pre-pubertal female mice activate TAp63 *via* ATM and Chk2 kinases (Suh et al., 2006; Livera et al., 2008; Gonfloni et al., 2009; Bolcun-Filas et al., 2014; Tuppi et al., 2018). This then activates Puma and Noxa thereby triggering primordial follicle apoptosis, depletion of the follicular pool and infertility (Kerr et al., 2012). Thus, extensive DNA damage to oocytes within primordial follicles triggers follicular apoptosis (Figure 1B) and severely threatens the ovarian reserve.

Severe depletion of the primordial follicle pool leading to menopause before 40 years of age – at least 10 years earlier than the average age of the menopause – is known as premature ovarian insufficiency (POI; Webber et al., 2016). A less severe form of accelerated decline in ovarian reserve is premature ovarian aging (POA), also known as occult POI (Gleicher et al., 2011). POI and POA can be brought on by gonadotoxic treatment such as chemotherapy but in most cases, the underlying cause is unknown.

Both primordial and fully-grown oocytes from reproductively aged females exhibit higher levels of DNA breaks than young oocytes (Titus et al., 2013). Notably, these physiological levels of damage seen with aging are substantially less severe than those induced by exogenous noxious agents like chemotherapy (Subramanian et al., 2020). Collectively, this suggests that physiological levels of primordial oocyte injury – as opposed to unusually severe drug- or radiation-induced damage – do not necessarily trigger TAp63-mediated apoptosis. Furthermore, this milder damage can persist throughout folliculogenesis and oocyte growth, culminating in damaged fully-grown oocytes (see Figure 1C). It is entirely plausible that primordial follicles with low levels of damage that escape apoptosis can persist to the antral stage since TAp63 expression wanes at more advanced stages of follicular development (Suh et al., 2006). In keeping with this, high irradiation doses (0.45 Gy) fully activate TAp63 causing almost complete annihilation of the follicular pool, whereas most primordial follicles survive and TAp63 is only partially activated by milder degrees of injury induced by low doses of irradiation (0.1 Gy; Suh et al., 2006).

The combination of increased ROS and compromised DNA repair capacity associated with reduced expression of DNA repair genes predispose to DNA damage in aged oocytes (Titus et al., 2013; Winship et al., 2018). This has two major consequences, firstly, damage-induced attrition of the follicular pool due to TAp63-mediated primordial follicle depletion and, secondly, persistence of low levels of DNA damage throughout oocyte growth. Such damaged fully-grown oocytes could potentially mature into fertilizable eggs thereby posing risks to future offspring.

## Setx is Critical for Repairing Physiological Levels of Aging-Induced DNA Damage

Since aging predisposes to DNA damage in oocytes, any additional compromise to DNA repair capacity would be expected to exacerbate damage and accelerate depletion of the follicular pool. This model based on DNA damage therefore provides at least one mechanistic basis for POI and POA (Turan and Oktay, 2020).

We recently studied female mice carrying either heterozygous (*SETX*^+/−^) or homozygous (*SETX*^−/−^) deletion of *SETX*. We found that at young ages, neither follicle-enclosed oocytes within ovaries nor isolated fully-grown oocytes from *SETX* mutants exhibited increased DSBs compared with wild-type mice (Subramanian et al., 2020). Moreover, young mutant females produced normal yields of fully-grown oocytes and these oocytes underwent maturation with normal spindle assembly and chromosome segregation indistinguishable from wild-type oocytes. Thus, at young ages, loss of Setx has no detrimental effects showing that Setx is dispensable in oocytes, at least at young ages. This contrasts with males in which, loss of Setx induces complete sterility due to failure to repair DSBs induced during reciprocal recombination (Becherel et al., 2013). Exactly why oocytes are able to complete repair of recombination-induced DSBs but spermatocytes cannot remains to be determined.

We then studied aging female mice bearing in mind that female mice typically exhibit overt signs of reproductive aging from around 12 months of age when both oocyte numbers and developmental competence decline markedly (Pan et al., 2008; Greaney et al., 2018; Iljas et al., 2020). Using the DSB marker, γH2AX, we found that by 8 months of age, there were low levels of damage in *SETX*^+/+^ ovaries as expected (Subramanian et al., 2021). In contrast, follicle-enclosed oocytes within *SETX*^−/−^ ovaries were already showing over 3-fold higher levels of damage. Moreover, 8-month-old *SETX*^−/−^ follicles showed similarly elevated levels of TUNEL staining (Subramanian et al., 2021) altogether indicating that in the absence of Setx, oocytes within ovaries prematurely accumulated DNA damage that triggered apoptosis. Entirely in keeping with premature depletion of the ovarian reserve, 8-month-old *SETX*^−/−^ ovaries contained less than half the numbers of all follicle stages seen in *SETX*^+/+^ ovaries and produced less than half the numbers of fully-grown oocytes following hormonal priming (Subramanian et al., 2021). Collectively, therefore, these data show that in oocytes, Setx’s role is restricted to aging and prevents early-onset accumulation of DNA damage that would otherwise induce premature depletion of the ovarian reserve.

At 4 months of age, isolated fully-grown *SETX*^+/+^ and *SETX*^−/−^ oocytes had low levels of DNA damage (Subramanian et al., 2020, 2021). This remained largely unchanged at 8 months of age for *SETX*^+/+^ oocytes contrasting sharply with a 3–4-fold higher increase in *SETX*^−/−^ oocytes consistent with the increased damage observed at this age in growing follicle-enclosed *SETX*^−/−^ oocytes. Importantly, these data are the first to clearly demonstrate that physiological levels of damage brought about by natural aging – as opposed to extreme levels of damage induced experimentally by noxious agents – can, and does, persist throughout the growth stage of oocytes resulting in damaged fully-grown oocytes.

In summary, loss of Setx results in premature accumulation of DNA damage in oocytes during aging. Since damage is minimal in young *SETX*^−/−^ oocytes, increased damage in older *SETX*^−/−^ oocytes is not due to loss of Setx *per se* but the consequence of superimposed aging-related phenomenon. Given Setx’s role in DNA repair, it is very likely that during aging, oocyte DNA acquires DSBs that are constantly being repaired by Setx-dependent mechanisms.

Due to the direct relationship between aging and oxidative stress, and Setx’s importance in repairing oxidative stress-induced damage (Suraweera et al., 2007), we studied an *in vitro* model of increased oxidative stress (Subramanian et al., 2020). We found that long-term *in vitro* culture markedly increased ROS levels in oocytes (Subramanian et al., 2020). Importantly, young *SETX*^−/−^ oocytes with inherently low levels of damage, acquired increased damage after ROS levels had increased by the end of prolonged *in vitro* culture whereas *SETX*^+/+^ oocytes showed minimal damage. Significantly, preventing ROS increase by co-culture in the antioxidant, N-acetyl cysteine, prevented increased damage in young *SETX*^−/−^ oocytes (Subramanian et al., 2020). This strongly supports that Setx is critical for repairing ROS-induced damage in oocytes. Furthermore, we showed that ROS levels are increased *in vivo* in *SETX*^−/−^ oocytes after 8 months of natural aging (Subramanian et al., 2020).

**Figure 2:**
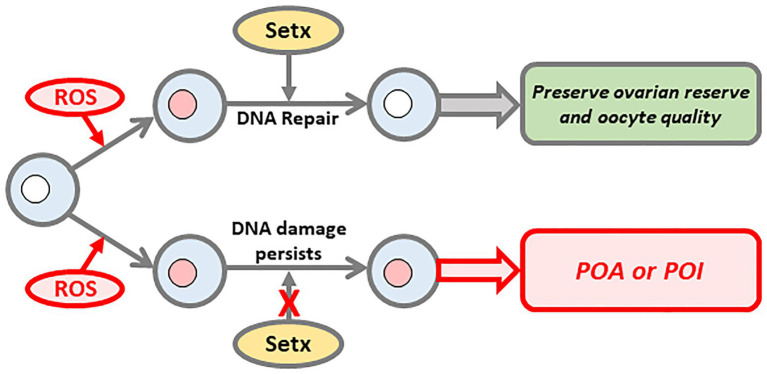
Senataxin (Setx) promotes DNA repair to preserve ovarian reserve and oocyte quality. During their protracted arrest in the ovary, oocytes are increasingly exposed to aging phenomenon such as oxidative stress, which induces DNA damage. Setx is required to repair this damage thereby preserving both oocyte number and quality (upper pathway). In contrast, on a background of compromised Setx function, DNA damage persists ultimately resulting in premature depletion of the oocyte pool and decline in oocyte quality (lower pathway).

Altogether, these data indicate that age-induced increases in oxidative stress constantly induce DNA damage that Setx-related mechanisms police and repair (Figure 2). We reason that by 12 months of age in mice, further increases in oxidative stress combined with compromised repair capacity ultimately tip the balance resulting in the higher basal levels of damage seen by this age in wild-type mouse oocytes (Titus et al., 2013).

## The *Setx* Mutant Model Provides New Insight Into *in Vivo* Acquired DNA Damage That Persists Throughout Oocyte Growth

### The SETX Model for Studying Persistent Oocyte DNA Damage

The foregoing indicate that damage acquired *in vivo* can trigger primordial follicle apoptosis but that in some cases, follicles escape apoptosis leading to damage that persists throughout oocyte growth leading to damaged fully-grown oocytes (Figure 1C). While the molecular basis underpinning primordial follicle apoptosis has been extensively investigated *via* experiments involving exogenously induced injury, little was known about how fully-grown oocytes respond to physiological damage that had persisted throughout the oocyte’s growth stage. The latter type of injury cannot readily be replicated by exogenous treatments since this typically produces severe levels of damage that trigger apoptosis (Figure 1B). Older *SETX* mutant females in which milder damage arises in oocytes, and persists throughout growth, therefore provides a very powerful model that not only enabled this to be definitively characterized as a distinct form of injury but also to investigate related molecular mechanisms for the first time. A major question was whether fully-grown oocytes that acquired damage *in vivo* could mount a cell-cycle response that would block meiotic maturation; this was a critically important question because if oocytes lacked this so-called DNA damage checkpoint, this could potentially result in the production of embryos with damaged DNA that would jeopardize pregnancy outcome. There is preliminary evidence in humans that oocyte DNA damage may well influence reproductive potential since women undergoing IVF treatment who produce oocytes with high levels of DSBs appear to have reduced pregnancy success rates (Astbury et al., 2020).

### The Molecular Basis of Oocyte Prophase Arrest and Oocyte Maturation

Before delving further into cell-cycle responses of *in vivo*-damaged oocytes, it will first be important to outline some key regulatory pathways. Oocytes within follicles are arrested at the dictyate stage of prophase, which, from a cell-cycle point of view, is equivalent to a late G2-phase (Solc et al., 2010; Adhikari and Liu, 2014; Greaney et al., 2018). Dictyate-arrested oocytes are identifiable by the presence of an intact nucleus known as the germinal vesicle (GV; Solc et al., 2010; Greaney et al., 2018). Oocyte maturation is initiated by activation of the master cell-cycle regulator, cyclin-dependent kinase 1 (Cdk1; also known as maturation promoting-factor or MPF), a heterodimer comprised of a catalytic Cdk1 subunit and a cyclin B1 co-activator. First meiotic maturation (MI) begins with GV breakdown (GVBD) marking entry into M-phase of MI and concludes with an extremely asymmetric division marked by extrusion of a very small first polar body (PB) into which half of the oocyte’s chromosomes are segregated (Greaney et al., 2018; Wei et al., 2018).

Maintenance of G2-arrest during the oocyte’s protracted prophase arrest (lasting up to 4–5 decades in humans) therefore revolves around preventing Cdk1 activation. This occurs *via* inhibitory phosphorylation of Cdk1 by Wee1/Myt1 kinases (Wee1B in oocytes), which is counteracted by Cdc25 phosphatases (Han et al., 2005; Solc et al., 2010; Adhikari and Liu, 2014; Adhikari et al., 2016). Critically, in oocytes, Cdk1 inhibition also involves proteolysis of the Cdk1 activator, cyclin B1, orchestrated by the Cdh1-activated anaphase-promoting complex (APC-Cdh1; Holt et al., 2011; Homer, 2013). Hence, prophase arrest is dependent upon Wee1B and APC-Cdh1 and is counteracted by Cdc25B.

Oocytes within follicles experience strong Cdk1 suppression due to Wee1B-mediated inhibition, which is further reinforced by inherently low levels of Cdk1 and cyclin B1 in incompletely grown oocytes (Kanatsu-Shinohara et al., 2000). Oocytes within antral-stage follicles have become fully grown and now contain adequate levels of cyclin B1 to support Cdk1 activation provided inhibitory Wee1B-mediated Cdk1 inhibition can be lifted. Release from Wee1B-induced inhibition is induced by the surge of luteinizing hormone (LH), which leads to suppression of Wee1B and activation of Cdc25B culminating in Cdk1 activation (Solc et al., 2010). The LH surge, therefore, triggers ovulation as well as oocyte maturation leading to the release of a mature metaphase II (MII)-arrested oocyte (or egg) capable of undergoing fertilization.

### Identification of a Novel DNA Damage Response in Oocytes

In somatic cells, DNA damage at G2-phase triggers a checkpoint *via* the DNA damage response (DDR), which delays entry into M-phase thereby enabling DNA to be repaired (Harper and Elledge, 2007; Carroll and Marangos, 2013). The canonical DDR is a phosphorylation cascade involving the apical kinases, ATM, and ATR, as well as downstream checkpoint kinases, Chk1/Chk2 (Harper and Elledge, 2007; Carroll and Marangos, 2013). The latter result in inhibition of Cdc25 phosphatase thereby preventing Cdk1 activation. Thus, the canonical DDR is a phosphorylation-centerd response that blocks entry into M-phase by preventing activation of the Cdk1 activator, Cdc25B.

To determine whether damaged fully-grown oocytes mount a DDR, the standard approach has been to isolate fully-grown oocytes with intact DNA from young female mice and to subject these isolated GV-stage oocytes to exogenous treatments such as chemotherapy or radiation *in vitro* (Figure 1D; Marangos and Carroll, 2012; Collins et al., 2015; Marangos et al., 2015; Collins and Jones, 2016; Mayer et al., 2016). Surprisingly, and in contrast to somatic cells, oocytes carrying severe damage induced by such treatments failed to undergo a G2-arrest and readily progressed into M-phase (Figure 1D; Marangos and Carroll, 2012; Collins et al., 2015; Marangos et al., 2015; Collins and Jones, 2016; Mayer et al., 2016). From this it was inferred that oocytes may lack a checkpoint at the G2-M boundary (Marangos and Carroll, 2012). It appears that the inability to respond to sudden induction of injury is due to an inherently ineffectual ATM-mediated phosphorylation pathway (Marangos and Carroll, 2012). Intriguingly, although entry into M-phase was not impaired by DNA damage, oocytes subsequently arrested in M-phase due to activation of the spindle assembly checkpoint (SAC) thereby preventing completion of MI (Collins et al., 2015; Marangos et al., 2015; Collins and Jones, 2016). The SAC incorporates key players such as Mad2 and BubR1 (encoded by *BUB1B*) and prevents chromosome mis-segregation by delaying anaphase-onset in the presence of chromosomes that have not become properly attached to spindle microtubules (Musacchio and Salmon, 2007; Homer, 2011). Not surprisingly, poorer quality oocytes from aged women and mice characterized by high aneuploidy rates have reduced expression of *MAD2* and *BUB1B* (Steuerwald et al., 2001, 2007; Pan et al., 2008; Riris et al., 2014). Thus, acutely induced DNA injury does not trigger a DDR at the G2-M boundary in oocytes but activates the SAC during M-phase.

It is important at this stage to revisit salient characteristics of *in vivo* acquired DNA damage in fully-grown oocytes and how it differs from acute damage induced experimentally *in vitro*. Firstly, the extent of *in vivo* damage is very mild compared to that induced by drug or radiation treatment (Figures 1C,D); had it been severe, primordial follicle apoptosis would have been triggered thereby pre-empting the emergence of fully-grown oocytes (Figure 1B). Secondly, and highly significantly, *in vivo* damage persists for a prolonged period during the oocyte growth phase (Figure 1C) – lasting 2–3 weeks in mice and 2–3 months in humans (Gosden and Lee, 2010; Greaney et al., 2018) – whereas *in vitro* induced damage is acute and short-lived (Figure 1D). Since *in vivo* exposure to injurious agents (e.g., oxidative stress; environmental toxins) occurs over prolonged periods throughout the individual’s lifetime, it is highly implausible for an oocyte to be completely free of damage throughout its entire growth phase within ovarian follicles *in vivo* and then to suddenly acquire very severe damage only at the very transient period when ovulation is about to occur. Previous experimental models involving short-term *in vitro* treatment of DNA-intact fully-grown oocytes (Figure 1D) do not therefore replicate the longer-term pattern of DNA damage occurring *in vivo*. In contrast, *SETX* mutant oocytes acquire modest levels of damage *in vivo* brought on by age-related increases in oxidative stress and not because of sudden exposure to artificial treatment (Subramanian et al., 2020). *SETX* mutant oocytes therefore provided a unique opportunity to study fully-grown oocytes that have acquired comparatively mild levels of DNA damage *in vivo*.

Using this model, we unexpectedly found that GVBD rates in *SETX*^−/−^ oocytes from 8-month-old females, which possessed increased DNA damage, was severely reduced (Subramanian et al., 2020). This indicated that *in vivo* acquired DNA damage does induce an arrest at the G2-M boundary (Figure 1C). Because artificially induced short-lived injury in fully-grown oocytes does not impair the G2-M transition (Figure 1D; Marangos and Carroll, 2012; Collins et al., 2015; Marangos et al., 2015; Collins and Jones, 2016; Mayer et al., 2016), this suggested that a longer duration of injury may be critical for allowing this unique DDR to be mounted in oocytes. This was indeed the case since the same *in vitro* chemotherapy-induced damage, which did not impair meiotic maturation immediately after damage induction, did lead to a robust G2-arrest if a delay was imposed following chemical treatment (Subramanian et al., 2020).

Intriguingly, we found that this novel delayed-response DDR in oocytes does not suppress Cdk1 activation *via* canonical phosphorylation-related pathways but by increased APC-Cdh1-mediated proteolysis of cyclin B1 (Subramanian et al., 2020). Increased APC-Cdh1 activity in turn is the consequence of increased activity of the APC-Cdh1 activator, Cdc14B, and reduced levels of the APC-Cdh1 inhibitor, Emi1 (Subramanian et al., 2020).

## Premature Decline in Fertility and Acute Sensitivity of Oocytes to Setx Compromise

The foregoing showed that in mice, compromised *SETX* function led to premature increases in oocyte DNA damage *in vivo* and to early-onset reduction in ovarian follicle numbers. We then asked how this might impact fertility during natural aging. For the first 6 months of a 12-month-long breeding trial, mean litter sizes and cumulative numbers of pups produced by *SETX*^−/−^ and wild-type females were indistinguishable (Subramanian et al., 2021). From 7 months of age onwards, however, coinciding with the observed increase in *in vivo* DNA damage in *SETX*^−/−^ oocytes, *SETX*^−/−^ females produced significantly fewer pups than wild-types and this difference was sustained throughout the remainder of the trial to 13 months of age (Subramanian et al., 2021). This decline in fertility was due to reduced numbers of fully-grown oocytes as well as reduced oocyte quality manifested as failure of maturation. Thus, compromised Setx function predisposes to aging-induced DNA damage, which leads to a premature decline in female fertility due to reduced ovarian reserve and compromised oocyte quality.

We also studied heterozygous females carrying one mutated *SETX* gene (*SETX*^+/−^ females) and surprisingly found that all reproductive parameters – oocyte DNA damage, follicle numbers, follicle atresia, numbers of fully-grown oocytes, oocyte maturation, and litter sizes – in 8-month-old *SETX*^+/−^ females were equally impaired as in 8-month-old *SETX*^−/−^ females (Subramanian et al., 2021). Further investigation identified that Setx expression from the remaining intact *SETX* gene in *SETX*^+/−^ oocytes could only marginally compensate for the missing gene (Subramanian et al., 2021) indicating that both genes are simultaneously required to sustain adequate function in oocytes. Interestingly, Setx expression in somatic cells from *SETX*^+/−^ females was significantly higher than in *SETX*^+/−^ oocytes (Subramanian et al., 2021) pointing to an unusually high sensitivity of oocytes to Setx deregulation. The vulnerability of oocytes to Setx dysfunction suggests that females with heterozygous *SETX* mutations unlikely to produce an overt somatic phenotype could nevertheless be acutely vulnerable to POA making *SETX* one of the very few genes associated with silent POA.

## Summary and Conclusion: Implications for Understanding POA in Humans

Although POA affects roughly 10 times more women than POI much less is known regarding POA’s genetic causation. Gaining increased understanding about genetic causes of POA is particularly challenging since, unlike POI, which has overt manifestations and diagnostic criteria (Webber et al., 2016), POA often only comes to light if women seek fertility treatment. Our studies in mice show that *SETX* mutations produce a model of POA involving DNA damage secondary to compromised DNA repair capacity. Agents capable of inducing damage which accumulate with aging such as oxidative stress, pose a constant threat requiring robust Setx-dependent DNA repair mechanisms to maintain DNA integrity in oocytes (Figure 2). Any inherent threat to such repair capacity as with *SETX* mutations tips the balance toward damage at earlier ages with severe consequences for the ovarian reserve (Figure 2).

Importantly, new data now show that oocytes mount a DDR against *in vivo* damage and that this is a slow-evolving response (Figure 1C; Subramanian et al., 2020). In contrast, damage that is experimentally induced *in vitro* is an acute event, against which, oocytes are unable to mount a DDR (Figure 1D). However, the physiological relevance of the latter is questionable since it would require an *in vivo* scenario in which, only fully-grown oocytes within large antral follicles are exposed to a damage-inducing event during the transient peri-ovulatory period.

Oocytes appear to be especially dependent on Setx since loss of function from even one gene in mice has consequences equally as severe as loss of both genes. Since the best-known human disorder resulting from compromised Setx function, AOA2, involves homozygous mutations (Lavin et al., 2013), heterozygous Setx compromise may well be a cause of seemingly idiopathic POA. It is important to stress that we identified a link between *SETX* mutation and POA rather than the more severe end of the spectrum, POI. *SETX* mutant ovaries initially contained normal numbers of follicles and produced normal numbers of fully-grown oocytes in young adulthood but subsequently experienced accelerated follicular depletion (Subramanian et al., 2020, 2021). In contrast, mutation of *ATM*, which causes another ACRA, A-T, in humans (Savitsky et al., 1995), induces POI with almost all follicles becoming depleted within a few days after birth in mice (Di Giacomo et al., 2005).

Investigating the role of *SETX* in POA in humans may not be straightforward due to the pleiomorphic effects of *SETX* mutations. Indeed, different *SETX* mutations cause up to four distinct neurological disorders (Lavin et al., 2013). It is therefore likely that not all *SETX* mutations impact ovarian aging in the same way and that different mutations may have distinctly different effects. Indeed, there has been a case-report of an 18-year-old female with AOA2 caused by a rare non-coding mutation who had polycystic ovarian syndrome (PCOS), a condition characterized by *high* ovarian reserve (Fogel et al., 2009). We note as well that AOA2 is caused by a plethora of different mutations (Anheim et al., 2009; Nanetti et al., 2013) whereas in our mouse model, the *SETX* gene was disrupted by a single mutation – a deletion of exon 4 – which resulted in a complete absence of Setx protein in knockout animals (Becherel et al., 2013).

Premature ovarian aging remains an enigmatic condition with very few known genetic causes. Very recent studies in the mouse model suggest that *SETX* mutations can induce a POA phenotype. Based on the variability in neurological disease profile produced by different *SETX* mutations in humans, it may well be that only a fraction of mutations results in POA. Identifying which *SETX* mutations are linked with POA in women will be an important future undertaking.

## Author Contributions

The author confirms being the sole contributor of this work and has approved it for publication.

### Conflict of Interest

HH is a co-founder, shareholder, and advisor of Jumpstart Fertility Inc., which was founded to develop research into NAD+-dependent pathways involved in female fertility.
